# Investigation of the Relationship between Oral Human Papillomavirus Infection and Oral Health

**DOI:** 10.30476/DENTJODS.2021.88551.1338

**Published:** 2022-03

**Authors:** Massoumeh Zargaran, Farid Azizi Jalilian

**Affiliations:** 1 Dept. of Oral and Maxillofacial Pathology, Faculty of Dentistry, Kurdistan University of Medical Sciences, Sanandaj, Iran; 2 Dept. of Medical Virology, Faculty of Medicine, Hamadan University of Medical Sciences, Hamadan, Iran

**Keywords:** Oral health, Human papillomavirus, Oral cavity, Infection

## Abstract

**Statement of the Problem:**

Oral health status has been suggested as one of the possible risk factors for oral human papillomavirus (OHPV) infection. There is inconsistent evidence concerning its relationship with the presence of OHPV.

**Purpose:**

This study aimed to compare oral health status between two groups of patients with and without OHPV infection.

**Materials and Method:**

This cross-sectional study was performed on 272 oral rinse samples, collected from our previous study population. After signing the written informed consents, the oral examination was performed to determine some clinical parameters of oral health status including periodontal disease status, oral hygiene status, decayed, missing, and filled teeth (DMFT) of participants. Next, viral deoxyribonucleic acid (DNA) extraction, polymerase chain reaction (PCR), and HPV genotyping tests were performed on the samples.

**Results:**

OHPV DNA was detected in a total 31 (11.40%) samples that were considered as OHPV+ group. None of the determined clinical parameters of oral health status was significantly different between OHPV+ and OHPV DNA negative (OHPV-) groups (*p*> 0.05).

**Conclusion:**

The findings of the current study did not indicate a significant association between oral health status and OHPV. Further studies with larger sample size are recommended to reach a definite conclusion.

## Introduction

Human papillomavirus (HPV) is a small, non-enveloped, epitheliotrophic, DNA virus [ 1 - 2 ]. HPV is classified into low and high-risk types, respectively causing benign and malignant epithelial lesions, such as head and neck squamous cell carcinomas (HNSCCs) [ 1 - 2 ]. The increased rate of HPV-related HNSCCs is attributed to the increased rate of infection with the virus [ 3 - 5 ]. Therefore, recognizing the risk factors for HPV infection may help identify high-risk individuals for HNSCCs and improve the associated mortality [ 1 ]. The oral health status has been suggested as one of the possible risk factors for oral HPV (OHPV) infection [ 6 ], which can be clinically examined through analysis of oral hygiene, periodontal disease, and decayed, missing, and filled teeth (DMFT) measurements [ 1 ].

Periodontitis is a chronic inflammatory disease [ 7 ], causing irreversible damage to the supporting structures of the teeth. It is clinically characterized by parameters, such as gingival inflammation, loss of tissue attachment, periodontal pocket formation, and alveolar bone loss [ 3 , 7 ]. Due to the continuous release of inflammatory cytokines and inflammatory biomarkers that continuously stimulate the proliferation of junctional epithelial basal cells [ 3 , 7 ] and increase the likelihood of aberrant DNA repair alongside with replication errors [ 3 ], patients with chronic periodontitis seem to be at an increased risk of HNSCCs [ 3 , 8 ]. Cellular proliferation, inflammation, and disturbance in the odontogenic tissue that lead to the progression of periodontal disease may be due to the infection of basal cells with OHPV [ 9 ], as periodontal pockets may serve as a reservoir for the virus [ 7 ].

Poor oral hygiene is another factor that facilitates the penetration of OHPV into the epithelium and causes infection in basal cells by inducing inflammation in the gingival mucosa and microscopic lesions [ 6 ]. It seems that individuals with poor oral hygiene have an unfavorable DMFT index [ 10 ] and are exposed to a higher risk of HNSCCs [ 3 ]. However, there is scarce and inconsistent evidence regarding the relationship of these clinical parameters with the presence of OHPV [ 1 - 2 , 9 , 11 - 12 ].

Studies by Ortiz *et al*. [ 9 ] and Wiener *et al*. [ 2 ] reported an association between OHPV and periodontitis, while Sun *et al*. [ 1 ], Horewicz *et al*. [ 11 ], and Jacob *et al*. [ 12 ] did not find a significant relationship. In the latter two studies [ 11 - 12 ], no virus was detected in the samples of groups with a healthy periodontium and chronic periodontitis. In the study by Sun *et al*. [ 1 ], the DMFT index and oral health status were not associated with OHPV infection, whereas Bui *et al*. [ 13 ] and Dalla Torre *et al*. [ 6 ] reported a significant relationship between poor oral hygiene and the presence of OHPV.

The current study aimed to compare the oral health status in terms of oral hygiene, periodontal disease status, and DMFT index between two groups of participants with and without OHPV infection.

## Materials and Method

### Patients

This descriptive, analytical, cross-sectional study was performed on 272 oral rinse samples, collected from our previous study population, presenting to the dental clinic of the Faculty of Dentistry of Kurdistan University of Medical Sciences between January 1 and November 1, 2018 [ 14 ]. All participants signed a written informed consent. The eligibility indicators were as follows: age of at least 18 years; not having cancer, lesions associated with OHPV infection, or severe systemic diseases [ 1 , 14 ]; and having at least 20 teeth to study the correlation between periodontal disease and OHPV infection [ 1 ]. The age and sex of the participants were documented in this study. In order to evaluate the represented clinical parameters of oral health status such as periodontal disease status, oral hygiene status, and DMFT index, oral and dental examinations were performed by a specialist dentist based on the study by Sun *et al*. [ 1 ]. 

The dental plaque, supragingival calculus, and subgingival calculus levels were classified as mild (identified in <30% of all teeth), moderate (identified in 30-60% of all teeth), and severe (identified in >60% of all teeth). To determine the periodontal disease status, the periodontal screening record was used. For this purpose, all six anatomical sextants in the mouth were scored as zero , one (presence of BOP, lack of calculus, and depth of periodontal pocket probing <3.5mm), 2 (BOP and calculus and a probing depth <3.5 mm), 3(BOP and calculus, 3.5< probing depth <5.5mm), and 4( BOP and calculus and probing depth >5.5mm). 

Then, participants were classified into two groups, if any of the six periodontal screening record scores was >3 (periodontal disease group), or none of the six periodontal screening record scores were >2 (nonperiodontal disease group), respectively. Regarding the oral hygiene, any plaque or supra/subgingival calculus score above moderate was considered as poor/fair (versus good/excellent) [ 1 ]. The DMFT index of each participant was determined by calculating the number of decayed, missing, and filled teeth and recorded in one of the following subgroups: 0-9, 10-19, and 20-28 [ 1 ].

### Collecting samples and extracting viral DNA

Samples of oral rinse (by swishing and gargling 10mL normal saline) were collected in a 50mL Falcon tube. Then, collected samples were transferred to the virology laboratory and stored at -20°C until use. After thawing, 5mL of all samples were centrifuged at 3000 rpm for15 minutes. The supernatant was discarded, and the pellets resuspended in 500µl of phosphate buffer solution (PBS). Briefly, extraction of viral DNA was carried out by a highly pure viral nucleic acid kit (Roche, Switzerland). We extracted the DNA from 200 μl of sample and eluted in 50μL of Elution Buffer. Finally, the extracted DNA was maintained at -20°C for further investigations [ 14 ].

### Polymerase chain reaction (PCR) assay and HPV genotyping

The extracted DNA was amplified by a frequently used kit according to guidelines published by the producer. In summary, a reaction contains 14.7 µl of HPV PCR mix, 0.3µl of Taq DNA polymerase, and 5µl of the template was amplified with a denaturation step at 94°C (for 3 minutes), 40 cycles of the 30s at 94°C, 30s at 55°C, 30s at 72°C and a final extension at 72°C (for 5minutes). Subsequently, PCR products were exposed to 2% agarose gel electrophoresis. For qualitative detection and identifying 32 HPV types, INNO-LiPA HPV Genotyping Extra II (Fujirebio, Japan) was employed. In summary, following the producer's guidelines, we amplified the part of the L1 region (SPF10 region). The next step was denaturation and hybridization of PCR products on a single-typing strip coated with HPV- and internal control-specific oligonucleotide probes. The process comprised of incubating, stringent rinsing, and manual color development. The interpretation of strips was performed manually according to a guideline [ 14 ].

### Statistical analysis

The participants were divided into two groups of positive and negative OHPV DNA in oral rinse samples (OHPV+ and OHPV-). Chi-square and Fisher’s exact tests were applied for comparing factors and parameters of both groups, using statistical package for social science (SPSS) version 22. The *p* Value< 0.05 was considered significant.

## Results

In total 141 (51.8%) men and 131 (48.2%) women, with the mean age of 34.9528.237 years (ranging from 19 to 60) were investigated. The frequency of dental plaques, supragingival calculus and subgingival calculus levels as well as periodontal disease, oral health status, and DMFT of the participants are presented in Table 1. Out of 272 samples, 31 (11.40%) and 241 (80.60%) were OHPV+ and OHPV-, respectively. However, only two HPV genotypes (HPV6 and HPV53) were detected in two samples. Table 2 presents the frequency of the studied parameters separately for OHPV+ and OHPV- groups. There was no significant difference between the two groups in terms of dental plaque, supragingival calculus, and subgingival calculus levels (*p*> 0.05). Periodontal disease status (*p*= 0.593), oral health status (*p*= 0.611) and DMFT (*p*= 0.517) were also not significantly associated with the presence of OHPV infection.

**Table 1:** Prevalence of studied variables in participants

Variable	Total number (%)
Dental plaque level	
-	7(2.6)
Mild	120(44.1)
Moderate	106(39.0)
Severe	39(14.3)
Supragingival Calculus level	
-	49(18.0)
Mild	150(55.1)
Moderate	63(23.2)
Severe	10(3.7)
Subgingival calculus level	
-	161(59.2)
Mild	90(33.1)
Moderate	17(6.3)
Severe	4(1.5)
Oral hygiene status	
Good to excellent	120(44.1)
Poor to fair	152(55.9)
DMFT	
0-9	125(45.9)
10-19	140(51.5)
20-28	7(2.6)
Periodontal status	
Non-periodontal disease	209(76.3)
Periodontal disease	63(23.2)

**Table 2:** Oral health clinical parameters and oral human papilloma virus status of participants

Variable	OHPV+(%)	OHPV-(%)	*p* Value	Test
Dental plaque				
-	0(0.0)	7(100.0)		
+	31(11.7)	234(88.3)	1.000	Fisher exact test
Dental plaque level				
-	0(0.0)	7(100.0)		
Mild	15(12.5)	105(87.5)		
Moderate	8(7.5)	98(92.5)		
Severe	8(20.5)	31(79.5)	0.121	*X^2^*
Supragingival calculus				
-	5(10.2)	44(89.8)		
+	26(11.7)	197(88.3)	0.772	*X^2^*
Supragingival calculus level				
-	5(10.2)	44(89.8)		
Mild	18(12.0)	132(88.0)		
Moderate	8(12.7)	55(87.3)		
Severe	0(0.0)	10(100.0)	0.679	*X^2^*
Subgingival calculus				
-	17(10.6)	144(89.4)		
+	14(12.6)	97(87.4)	0.600	*X^2^*
Subgingival calculus level				
-	17(10.6)	144(89.4)		
Mild	14(15.6)	76(84.4)		
Moderate	0.(0.0)	17(100.0)		
Severe	0(0.0)	4(100.0)	0.226	*X^2^*
Oral hygiene status				
Good to excellent	15(12.5)	105(87.5)		
Poor to fair	16(10.5)	136(89.5)	0.611	*X^2^*
DMFT				
0-9	13(10.4)	112(89.6)		
10-19	18(12.9)	122(87.1)		
20-28	0(0.0)	7(100.0)	0.517	*X^2^*
Periodontal status				
Non-periodontal disease	25(12.0)	184(88.8)		
Periodontal disease	6(9.5)	57(90.5)	0.593	*X^2^*

## Discussion

The relationship between OHPV infection and oral health status has been evaluated objectively in only a few studies, the results of which are contradictory [ 1 - 2 , 6 , 9 , 11 - 13 ]. Therefore, the present study aimed to investigate this relationship.

Although bacteria are undoubtedly the main cause of periodontal disease, they are not always the main cause of the multiplicity of the clinical manifestations of this disease [ 15 ]. Therefore, other factors may also have significant impacts on the onset and progression of periodontitis [ 9 , 16 ]. Microorganisms may act as pathogens of periodontal disease with having three characteristics including the ability to colonize (entering and reproducing in the host cell), the ability to escape the host defense mechanisms; and the ability to produce substances that directly initiate tissue destruction. Some of these characteristics can be found in viruses, such as HPV [ 17 ]. Some studies have identified the presence of HPV in the periodontium and suggested a possible relationship between periodontitis and HPV [ 7 , 17 ], however, in other studies, the virus was not detected in healthy or periodontal disease tissue samples [ 11 - 12 ]. In addition, the reported results are inconsistent regarding the relationship between periodontal disease and OHPV [ 1 - 2 , 9 ]. In contrast to studies by Wiener *et al*. [ 2 ] and Ortiz *et al*. [ 9 ], no significant relationship was observed between these variables in the present study; however, our results are in line with the study by Sun *et al*. [ 1 ]. Nevertheless, a wide range of mechanisms contributes to the biological plausibility of this relationship [ 17 ] described as follows.

First mechanism is defined as OHPV may be involved in the development of periodontitis [ 9 ] and trigger periodontal disease [ 2 ]. The junctional epithelium is a very thin, non-keratinized, permeable epithelium put into a humid environment that provides an appropriate condition for viral growth [ 15 ], resulting in the increased affinity of HPV [ 17 ]. HPV infection can cause some changes such as the increased cell proliferation of junctional epithelium [ 9 , 12 ] as the main hallmark of periodontal destruction [ 12 ], and its conversion to periodontal pockets as the hallmark of disease initiation [ 9 ]. Theoretically, it has been suggested that infections caused by viruses increase the susceptibility of periodontal tissues to lytic activity, destruction of tissue by the immune system, and suppression of the immune system, that in turn increase the host's vulnerability to bacterial infections [ 18 ].

The second mechanism is described as periodontitis can increase the acquisition and continuation of OHPV infection [ 9 , 16 ]. It may be caused by the inflamed periodontium of the periodontal pocket and its consequent pathological changes [ 9 ], such as transformation of the junctional epithelium to the stratified squamous epithelium, the emergence of the characteristics of continuous epithelial rapid increase, movement, creation of rete ridge, ulcers, the presence of integrin 6 4 and syndecan-1 (potential receptors of HPV) [ 16 ]. HPV infection of exposed basal cells within the periodontal pocket increases the proliferation of these cells [ 9 ] and enhances viral replication, viral shedding [ 9 ], and finally, viral load in the saliva [ 9 ]. 

The presence of viruses (such as HPV) and their interaction [ 19 ] alongside with the significantly higher HPV copy number in individuals with a high oral bacterial count [ 20 ] hypothetically may propose a risk increase of OHPV infection by increasing the number of oral bacteria. Teeth, fillings, restorations [ 21 ], and even dental calculi [ 22 ] are considered as harbors for microbiomes since microbes attach to them and form a microbial population (dental plaque) [ 21 , 23 ]. Excessive accumulation of dental plaque can cause periodontitis [ 9 ] and increase OHPV replication [ 20 ] through inflammation and enhancing the proportion of proteolytic and usually obligate anaerobic pathogens of periodontal disease [ 24 ]. 

In this study, similar to the study by Sun *et al*. [ 1 ], the prevalence of OHPV in individuals with dental plaque, regardless of the plaque levels, was higher than those without dental plaque were. Unlike the study by Dalla Torre *et al*. [ 6 ], who reported a significant relationship between dental plaque and the presence of OHPV, in the present study, in line with the study by Sun *et al*. [ 1 ], this parameter was not significantly different between the OHPV+ and OHPV- groups. Contrary to the present study and the study by Sun *et al*. [ 1 ], Dalla Torre *et al*. [ 6 ] showed that the percentage of OHPV+ cases increased by increasing the amount of dental plaque.

The consequence of bacterial plaque mineralization is dental calculus, which due to its rough surface, can both increase the aggregation of plaque and toxins produced by bacteria and preventing their removal [ 22 ]. Periodontal degradation and damage are associated with the presence of calculus, as the calculus facilitates further bacterial involvement [ 25 ]. Similar to Sun *et al*. [ 1 ], in the present research, the prevalence of positive OHP-V was more in the supragingival calculus+ and subgingival calculus+ groups compared to the negatives. However, in both studies, these variables were not significantly different between the OHPV+ and OHPV- groups.

Another factor that can lead to further microbial plaque accumulation and increase the risk of OHPV infection is poor oral hygiene [ 26 - 27 ]. In this study, as well as in the previous report [ 1 ], the status of oral hygiene in OHPV+ and OHPV- groups was not significantly different. However, this finding was inconsistent with the result of studies by Bui *et al*. [ 13 ] and Dalla Torre *et al*. [ 6 ], which indicated a considerable relationship between poor oral hygiene and the risk of OHPV infection. These contradictory findings may be attributed to different methodologies used to determine oral hygiene status. The clinical parameters and their measurements for the present study were similar to Sun *et al*. [ 1 ] but dissimilar to Dalla Torre *et al*. [ 6 ]. It should be noted that in the study by Bui *et al*. [ 13 ], the oral health status of the participants was determined based on their own self-report and was not measured objectively.

DMFT is one of the valuable parameters to determine the oral health status [ 10 ]. The DMFT index is one of the tools used to assess dental caries [ 28 - 29 ]. Since further microbial accumulation can result in more dental caries [ 23 ] and increase DMFT, it seems that this index can indirectly represent the amount of dental plaque accumulation. This index has also been reported to be more unfavorable in people who neglect their oral health [ 10 ]. In the present study, similar to the study by Sun *et al*. [ 1 ], there was no significant difference in terms of DMFT between the OHPV+ and OHPV- groups. 

Sun *et al*. [ 1 ], Dalla Torre *et al*. [ 6 ], and Wiener *et al*. [ 2 ] performed their studies on OHPV+ samples with specific genotypes, while in the present study, only two cases with genotypes were defined. Therefore, similar to the study by Ortiz *et al*. [ 9 ], all HPV DNA positive cases were included in this study to investigate the relationship between oral health and OHPV, which is one of the limitations of the current study. Another limitation is cross-sectional design of the study as OHPV infection was examined at one point in time so it was impossible to determine number of the detected infections might be eliminated or considerable as persistent or new infections. It is even possible that the negative cases in this study were OHPV+ in the past.

## Conclusion

In this study, we did not find a relationship between oral health status and infection related to OHPV. Considering the limitations of this study, which might have affected the results, we suggest further research with a larger sample size, other designs (e.g., prospective longitudinal studies), other methods of sample collection (e.g., pocket scraping or biopsy of periodontal tissues), and different measurements of parameters (e.g., assessment of the alveolar bone loss via radiography). We also suggest future studies on samples with known HPV genotypes. 

## Acknowledgement

The authors wish to thank Mr. Naser Reshadmanesh for statistical assistance in this study. The Research Ethics Committee of Kurdistan University of Medical Sciences approved this study (IR.MUK.REC.1397.036). This work was supported by Kurdistan University of Medical Sciences (grant number: 1397.036) and written consents were obtained from all the participants. 

## Conflict of Interest

The authors declare no conflicts of interest.
